# Non-linear associations and threshold effects of BRI, CI, and WHtR with grip strength in U. S. adults aged ≥20 years: a cross-sectional study

**DOI:** 10.3389/fnut.2025.1597065

**Published:** 2025-06-18

**Authors:** Sijia Yang, Kun Liao, Lu Zhou, Shengbo Zhang, Jianchao Wu

**Affiliations:** ^1^Department of Thyroid Diagnosis and Treatment Center, Zhuhai People’s Hospital (The Affiliated Hospital of Beijing Institute of Technology, Zhuhai Clinical Medical College of Jinan University), Zhuhai, China; ^2^Department of Breast Surgery Ward, Zhuhai People’s Hospital (The Affiliated Hospital of Beijing Institute of Technology, Zhuhai Clinical Medical College of Jinan University), Zhuhai, China; ^3^Zhuhai Clinical Medical College of Jinan University (Zhuhai People’s Hospital, The Affiliated Hospital of Beijing Institute of Technology), Guangzhou, China

**Keywords:** cross-sectional study, body measures, obesity, NHANES, muscle strength

## Abstract

**Background:**

Muscle strength is strongly associated with various physiological functions and health risks, with grip strength serving as a key indicator for its assessment. Currently, the relationship between novel obesity indices [Body Roundness Index (BRI), Conicity Index (CI), and waist-to-height ratio (WHtR)] and grip strength remains unclear. The current study aimed to investigate the non-linear/threshold relationships between BRI, CI, WHtR, and grip strength.

**Methods:**

A cross-sectional study design was adopted to analyze the data of 9,356 participants from the National Health and Nutrition Examination Survey (NHANES) conducted between 2011 and 2014. Researchers measured grip strength and calculated BRI, CI, and WHtR, while controlling for age, sex, ethnicity, and other covariates. Statistical analyses included linear regression, smooth curve fitting, and threshold effect models to evaluate non-linear/threshold relationships. The significance level was set at a *p* < 0.05, and 95% confidence intervals (CIs) were reported.

**Results:**

BRI, CI, and WHtR exhibited significant non-linear associations with grip strength. For BRI, values below 3.55 exhibited a strong positive effect on grip strength (*β* = 3.60, 95% CIs: 2.81–4.39), with weakened but persistent positive effects above this threshold (*β* = 0.24, 95% CI: 0.10–0.39). WHtR demonstrated a similar pattern, with a threshold set at 0.51: *β* = 62.46 (48.36–76.55) below and *β* = 6.47 (2.85–10.08) above. CI showed an inverted U-shaped relationship, shifting from positive (*β* = 15.87, 7.85–23.90) to negative (*β* = −9.98, −14.98 to −4.98, *p* < 0.01) at a threshold of 1.27.

**Conclusion:**

In U. S. adults, BRI, CI, and WHtR exhibited non-linear and threshold-dependent associations with grip strength, suggesting that these indices can help refine the assessment of muscle strength. The findings indicate that integrating these indices could enhance the accuracy of risk stratification for muscle dysfunction, particularly in individuals with central obesity. Longitudinal studies are needed to further validate the causal relationships underlying these associations.

## 1 Introduction

Muscle strength is a core determinant of functional status and lifespan and is closely associated with the incidence and mortality of health risks such as sarcopenia, metabolic syndrome, and cardiovascular diseases (1–3). As a representative indicator of muscle strength, grip strength is critically important in evaluating muscle function and serves as one of the key markers for diagnosing sarcopenia (4, 5). Sarcopenia is an aging-related syndrome characterized by a gradual decline in skeletal muscle mass and strength, often accompanied by reduced physical function. Its prevalence in Asian countries ranges from 5.5 to 25.7%, imposing a significant socioeconomic burden (6, 7). Studies have found that sarcopenia can increase the risk of fractures and falls among the elderly, potentially even leading to severe consequences and death (8). In clinical practice, accurately identifying the association between fat distribution and muscle strength is crucial for the early screening of sarcopenia.

When evaluating indicators related to muscle strength, traditional obesity indices such as Body Mass Index (BMI) and Waist Circumference (WC) have certain limitations in reflecting the relationship between body fat distribution and muscle strength (9, 10). Epidemiological studies have revealed conflicting associations between traditional indices and muscle strength; for instance, cross-sectional analyses have shown a positive correlation between BMI and grip strength (11). In contrast, abdominal obesity indices such as waist circumference exhibit a negative correlation with muscle strength—for example, the waist circumference is significantly larger among older adults with sarcopenia than those without sarcopenia (12). These contradictions highlight the limitations of BMI and waist circumference in capturing the complex relationship between fat distribution and muscle health.

In recent years, novel anthropometric indices such as BRI, CI, and WHtR have gradually attracted attention (13). BRI can be used to assess visceral fat (14). A study conducted in China found that, as a new anthropometric index, BRI is more effective than BMI and WHtR in detecting a cluster of cardiovascular and metabolic abnormalities in Chinese women (15). CI has been applied to the diagnosis of diabetes and hypertension, correlates with lipid levels, and has also been used to evaluate central fat (13, 16). WHtR, a common index for assessing central obesity, has demonstrated advantages in predicting cardiovascular diseases and other conditions (17). However, the relationships between these indices and muscle strength remain controversial, particularly regarding non-linear associations or threshold effects, which have not yet been clarified.

Currently, no study has systematically explored the non-linear associations and threshold effects between BRI, CI, WHtR, and grip strength in U. S. adult populations. This study proposes the following hypotheses: BRI and WHtR are positively correlated with grip strength below specific thresholds, with the strengths of these associations decreasing above these thresholds. CI shows an inverted U-shaped relationship with grip strength. The research objectives are as follows: to quantify the dose–response relationships between BRI, CI, WHtR, and grip strength; to identify inflection points where the direction or intensity of association changes; and to analyze heterogeneity across subgroups of age, sex, and ethnicity. To test these hypotheses, we analyzed NHANES data from 2011 to 2014 using the smooth curve fitting and threshold effect models, after adjusting for confounding factors such as age, sex, and chronic diseases. By systematically evaluating the non-linear associations between novel obesity indices and grip strength, this study aims to provide evidence for muscle function assessment, in particular, new indices for stratifying central obesity populations. Clarifying these relationships will deepen our understanding of the complex interactions between body composition and muscle health and offer new perspectives for sarcopenia screening.

## 2 Methods

### 2.1 Study design

This study adopted a cross-sectional study design to analyze the associations between BRI, CI, and WHtR, and muscle strength. The research utilized data from the NHANES 2011–2014. This survey employs a scientifically rigorous, stratified, multi-stage probability cluster sampling method, broadly covering populations from diverse regions, socioeconomic backgrounds, ethnicities, and age groups in the United States. This approach provides representative sample resources for the study and aids in comprehensively and objectively exploring the relationships between target variables. All data can be obtained on the official NHANES website.^1^

### 2.2 Study population

**Figure 1:**
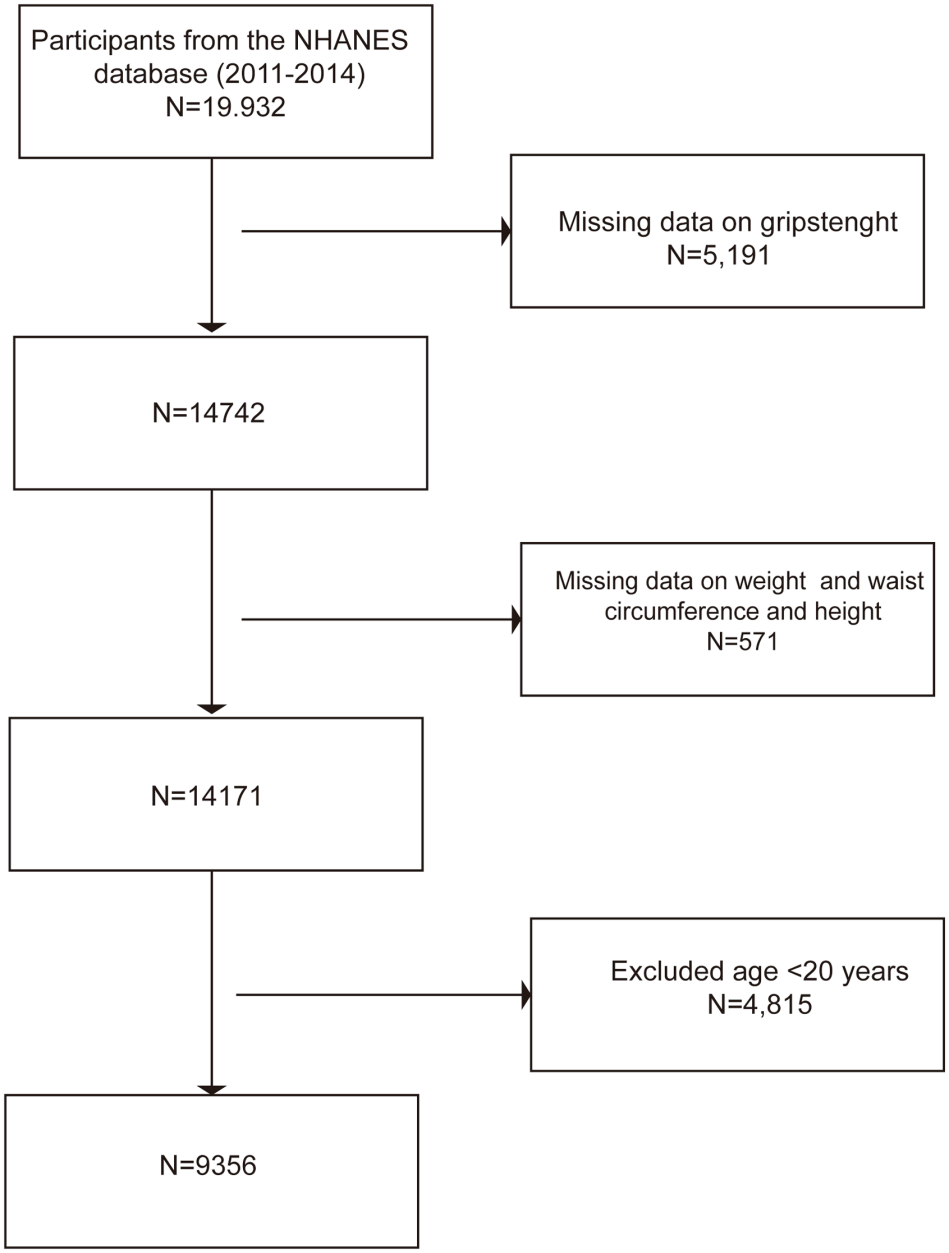
Flowchart of participant selection.

The data for this study were derived from NHANES 2011–2014, a nationally representative cross-sectional study. The inclusion criteria were participants aged ≥20 years with complete grip strength and anthropometric data (waist circumference, weight, height). The exclusion criteria were as follows: (1) age <20 years [according to the definition of the World Health Organization (WHO), adults are individuals over 19 years old (18), consistent with previous similar studies (19, 20)]; and (2) missing key variables (incomplete data on grip strength, waist circumference, weight, or height). The sample screening process is shown in Figure 1: initially, 19,932 participants were included, and those without grip strength data (*n* = 5,190), missing anthropometric data (*n* = 571), and those aged <20 years (*n* = 4,815) were excluded. Finally, the analysis sample included 9,356 adults.

## 3 Variable definitions and measurements

### 3.1 Assessment of grip strength

Grip strength measurement followed the NHANES standardized protocol (21), using a calibrated grip dynamometer (T. K. K. 5401, Takei Scientific Instruments, Japan). Participants stood upright with their arms naturally hanging down and their elbows fully extended and then tightly gripped the device with their dominant hand. Three consecutive measurements were taken for each hand at 60-s intervals, and the maximum value (in kg) was recorded as the final grip strength value, which is in line with muscle strength assessment standards (22).

### 3.2 Definition of BRI, CI, and WHtR

BRI: It is primarily used to measure body fat distribution. By combining height and waist circumference data, this index comprehensively reflects the overall obesity level of the human body and the fat distribution characteristics in the body, particularly providing a reference value for the assessment of visceral fat. Compared to traditional single indicators, it provides a more comprehensive reflection of the body’s fat accumulation status (14). The calculation formula is as follows:

$\mathrm{BRI}=364.2-365.5\times {\sqrt{1-(\mathrm{wc}(m)/2\times \pi \times \text{height}(m)/2)}}^{2}$

CI: CI is an index constructed based on the geometric characteristics of the body, which comprehensively considers factors such as weight, height, and waist circumference. It aims to overcome the limitations of the simple association between waist circumference and height and emphasizes the accumulation of abdominal fat. CI is of great significance in evaluating individual central obesity and related metabolic risks and is associated with multiple disease risks (23). The calculation formula is as follows:

$\mathrm{CI}=\frac{\mathrm{wc}(m)}{0.109\times \text{weight}(\mathrm{kg})\times \text{height}(m)}$

WHtR: WHtR measures body fat distribution by calculating the ratio of waist circumference to height. Its advantages include simple calculation and intuitiveness, which can effectively reflect central obesity and serve as a good indicator in predicting health risks such as cardiovascular diseases. It is one of the commonly used clinical indicators for rapidly assessing body fat distribution characteristics and potential health risks (13). The calculation formula is as follows:

$\text{WHtR}=\frac{\mathrm{wc}(m)}{\text{height}(m)}$

### 3.3 Covariates

The present study incorporated a series of covariates, including age, sex, ethnicity, alcohol consumption, hypertension, diabetes, special diet, total energy intake, coronary heart disease, marital status, and total cholesterol. These variables were selected based on their potential associations with muscle strength, obesity, or related physical indicators, to effectively reduce confounding bias. This approach was undertaken to achieve a more precise examination of the relationship between BRI, CI, WHtR, and muscle strength. The following definitions are provided for the selected covariates: Participants were asked if they consume alcohol and were divided into two groups accordingly: those who consumed alcohol and those who did not. In addition, participants were asked whether they had been diagnosed with hypertension, diabetes, or coronary heart disease by a physician. The Dietary Behavior questionnaire assessed current adherence to specialized diets (e.g., vegetarian and ketogenic), with such participants designated as “special dieters” (24). The total energy consumed by the participants through food and drink was calculated using the 24-h dietary review method. Laboratory detection module was used to collect the total cholesterol content (in mmol/L).

### 3.4 Statistical analysis

The normality of the grip strength variable was tested using multiple methods, including Anderson–Darling, Cramer–von Mises, Lilliefors (Kolmogorov–Smirnov), and Pearson’s chi-squared tests. The results indicated a significant deviation from the normal distribution (*p* < 0.001). Consequently, intergroup comparisons were performed using the Kruskal–Wallis H-test (for continuous variables) or Pearson’s chi-squared test (for categorical variables). Continuous variables are presented as the median (interquartile range), and categorical variables are reported as raw proportions.

The main analyses included multiple linear regression analyses of the associations between obesity indices (BRI, CI, and WHtR) and grip strength. The models were adjusted in three stages: Model 1 (unadjusted), Model 2 (adjusted for age, sex, and ethnicity), and Model 3 (additionally adjusted for chronic diseases, lifestyle, and total cholesterol). Non-linearity was assessed using restricted cubic splines (RCS), and the deviation of the curve from linearity was evaluated by the likelihood ratio test (*p* < 0.05). Threshold effect analysis utilized piecewise regression models to identify the inflection points (K) and to test significant differences in slopes on both sides. Subgroup analyses were stratified by age (20–39 years old, 40–60 years old, > 60 years old), sex, and ethnicity to explore heterogeneity. Interaction effects were evaluated by the likelihood ratio test (*p* < 0.05).

All analyses applied the NHANES complex sampling weights to ensure national representativeness. The significance level was set at a two-sided *p* < 0.05, and 95% confidence intervals (CIs) were reported. The analyses were completed using EmpowerStats 4.2 software.^2^

## 4 Results

### 4.1 Characteristics of the study population

Table 1 shows that a total of 9,356 participants were included in this study, with a balanced sex ratio (49.76% male and 50.24% female), and the median age was 47 years (ranging from 20 to 80 years). Non-Hispanic White individuals had the highest proportion in the racial distribution (40.79%). The quartile range of grip strength was 14.80 to 169.60 kg, the median energy intake was 3,864 kcal, the median CI was 1.31, the median BRI was 5.01, and the median WHtR was 0.58. The prevalence rates of hypertension, diabetes, and coronary heart disease were the highest in the first quartile group (Q1) with the lowest grip strength, at 45.29, 20.60, and 5.25%, respectively. The proportion of drinkers was the highest in the fourth quartile group (Q4), with the highest grip strength (87.82%). Factors such as education level, marital status, and special diet also showed significant differences among the grip strength quartiles (all *p* < 0.01).

**Table 1:** Basic characteristics of participants by grip strength quartile.

Characteristics	Overall	Grip strength quartile				*P*-value
		Q1 (14.80–54.20)	Q2 (54.30–67.40)	Q3 (67.50–87.50)	Q4 (87.60–169.60)	
*N*	9,356	2,325	2,352	2,339	2,339	<0.01
Age (years)	47.00 (20.00–80.00)	60.00 (20.00–80.00)	46.00 (20.00–80.00)	48.00 (20.00–80.00)	39.00 (20.00–80.00)	<0.01
Energy (kcal)	3864.00 (193.00–20050.00)	3547.00 (312.00–9318.00)	3824.00 (702.00–16562.00)	3864.00 (636.00–18959.00)	4356.00 (193.00–20050.00)	<0.01
Total cholesterol (mmol/L)	4.83 (1.53–21.02)	4.91 (1.53–21.02)	4.83 (1.78–15.83)	4.83 (2.12–9.93)	4.83 (2.07–13.52)	0.001
CI	1.31 (1.00–1.73)	1.32 (1.04–1.60)	1.30 (1.00–1.69)	1.31 (1.00–1.73)	1.29 (1.05–1.61)	<0.01
BRI	5.01 (1.05–23.48)	5.42 (1.41–23.48)	5.09 (1.17–18.59)	5.02 (1.05–18.79)	4.58 (1.27–20.30)	<0.01
WHtR	0.58 (0.36–1.14)	0.60 (0.38–1.14)	0.58 (0.36–1.02)	0.58 (0.36–1.03)	0.56 (0.37–1.06)	<0.01
Drink (%)						<0.01
Yes	7,052 (75.38%)	1,418 (60.99%)	1,697 (72.15%)	1883 (80.50%)	2054 (87.82%)	
no	2,303 (24.62%)	907 (39.01%)	655 (27.85%)	456 (19.50%)	285 (12.18%)	
Hypertension (%)						<0.01
Yes	3,290 (35.17%)	1,053 (45.29%)	749 (31.85%)	812 (34.72%)	676 (28.90%)	
No	6,065 (64.83%)	1,272 (54.71%)	1,603 (68.15%)	1,527 (65.28%)	1,663 (71.10%)	
Sex (%)						<0.01
Male	4,655 (49.76%)	196 (8.43%)	485 (20.62%)	1,661 (71.01%)	2,313 (98.89%)	
Female	4,700 (50.24%)	2,129 (91.57%)	1867 (79.38%)	678 (28.99%)	26 (1.11%)	
Ethnicity/Hispanic origin (%)						<0.01
Mexican American people	1,063 (11.36%)	277 (11.91%)	278 (11.82%)	252 (10.77%)	256 (10.94%)	
Other Hispanic people	870 (9.30%)	290 (12.47%)	213 (9.06%)	207 (8.85%)	160 (6.84%)	
Non-Hispanic White people	3,816 (40.79%)	979 (42.11%)	963 (40.94%)	866 (37.02%)	1,008 (43.10%)	
Non-Hispanic Black people	2,182 (23.32%)	349 (15.01%)	564 (23.98%)	620 (26.51%)	649 (27.75%)	
Other ethnicities - Including multiple ethnicities	1,424 (15.22%)	430 (18.49%)	334 (14.20%)	394 (16.84%)	266 (11.37%)	
Education level (%)						<0.01
Less than 9th grade	693 (7.41%)	257 (11.05%)	158 (6.72%)	177 (7.57%)	101 (4.32%)	
9–11th grade (includes 12th grade with no diploma)	1,273 (13.61%)	357 (15.35%)	281 (11.95%)	317 (13.55%)	318 (13.60%)	
High school graduate/GED or equivalent	2027 (21.67%)	499 (21.46%)	471 (20.03%)	479 (20.48%)	578 (24.71%)	
Some college or AA degree	2,931 (31.33%)	676 (29.08%)	786 (33.42%)	728 (31.12%)	741 (31.68%)	
College graduate or above	2,431 (25.99%)	536 (23.05%)	656 (27.89%)	638 (27.28%)	601 (25.69%)	
Marital status (%)						<0.01
Married	5,433 (58.08%)	1,195 (51.40%)	1,334 (56.72%)	1,415 (60.50%)	1,489 (63.66%)	
Widowed	622 (6.65%)	387 (16.65%)	138 (5.87%)	74 (3.16%)	23 (0.98%)	
Divorced	1,029 (11.00%)	315 (13.55%)	278 (11.82%)	236 (10.09%)	200 (8.55%)	
Separated	309 (3.30%)	88 (3.78%)	87 (3.70%)	79 (3.38%)	55 (2.35%)	
Never married	1962 (20.97%)	340 (14.62%)	515 (21.90%)	535 (22.87%)	572 (24.45%)	
Diabetes (%)						<0.01
Yes	1,358 (14.52%)	479 (20.60%)	319 (13.56%)	344 (14.71%)	216 (9.23%)	
No	7,997 (85.48%)	1846 (79.40%)	2033 (86.44%)	1995 (85.29%)	2,123 (90.77%)	
Other special diet (%)						<0.01
Yes	1,349 (14.42%)	389 (16.73%)	363 (15.43%)	314 (13.42%)	283 (12.10%)	
No	8,006 (85.58%)	1936 (83.27%)	1989 (84.57%)	2025 (86.58%)	2056 (87.90%)	
Coronary heart disease (%)						<0.01
Yes	330 (3.53%)	122 (5.25%)	68 (2.89%)	92 (3.93%)	48 (2.05%)	
No	9,024 (96.47%)	2,203 (94.75%)	2,283 (97.11%)	2,247 (96.07%)	2,291 (97.95%)	

Unadjusted model. *p < 0.05, **p < 0.01. BRI, Body Roundness Index; CI, Conicity Index; WHtR, waist-to-height ratio. Continuous variables are presented as the median (interquartile range), and categorical variables are expressed as raw proportions.

### 4.2 Associations between BRI, CI, WHtR, and grip strength

Table 2 presents the results of the multivariate linear regression analysis on the relationships between BRI, CI, and WHtR and grip strength. In the fully adjusted model (Model 3), there were significant positive linear associations between BRI, WHtR, and grip strength (BRI: *β* = 0.45, 95% CI: 0.30–0.60; WHtR: *β* = 11.94, 95% CI: 8.38–15.49), while CI did not show a significant linear association (*β* = −0.92, 95% CI: −4.69–2.85). The quartile analysis demonstrated that there was a dose–response relationship between BRI and grip strength, and the grip strength increased gradually with the increase in quartiles (Q2: *β* = 2.51, Q3: *β* = 2.71, Q4: *β* = 3.27; P for trend = 0.01). WHtR also indicated a significant increasing trend (P for trend = 0.01). In contrast, the quartiles of CI did not exhibit a consistent pattern (P for trend = 0.43). The above results indicate that increases in the levels of BRI and WHtR are positively correlated with the enhancement of grip strength, with stronger effects in the higher quartile groups. In contrast, no clear trend was observed in the relationship between CI and grip strength.

**Table 2:** Associations between BRI, CI, and WHtR and handgrip strength.

Characteristic	Model 1 [*β* (95% CI)]	Model 2 [*β* (95% CI)]	Model 3 [*β* (95% CI)]
BRI (continuous)	−1.02 (−1.21, −0.82)	0.42 (0.26, 0.58)	0.45 (0.30, 0.60)
BRI (quartile)			
Quartile 1	Ref.	Ref.	Ref.
Quartile 2	0.86 (−0.64, 2.37)	3.37 (2.33, 4.42)	2.51 (1.58, 3.45)
Quartile 3	−1.84 (−3.21, −0.46)	3.37 (2.33, 4.42)	2.71 (1.62, 3.80)
Quartile 4	−5.57 (−6.78, −4.36)	3.34 (2.24, 4.44)	3.27 (2.23, 4.30)
P for trend	<0.01	<0.01	0.01
CI (continuous)	−17.64 (−22.88, −12.40)	−0.34 (−4.43, 3.75)	−0.92 (−4.69, 2.85)
CI (quartile)			
Quartile 1	Ref.	Ref.	Ref.
Quartile 2	−0.54 (−2.10, 1.02)	2.01 (1.10, 2.92)	1.11 (0.30, 1.93)
Quartile 3	−2.09 (−3.51, −0.68)	1.95 (0.94, 2.95)	1.13 (0.20, 2.05)
Quartile 4	−4.21 (−5.56, −2.87)	−0.23 (−1.36, 0.90)	−0.43 (−1.48, 0.62)
P for trend	<0.01	0.58	0.43
WHtR (continuous)	−23.74 (−28.31, −19.18)	11.56 (7.65, 15.47)	11.94 (8.38, 15.49)
WHTR (quartile)			
Quartile 1	Ref.	Ref.	Ref.
Quartile 2	0.86 (−0.64, 2.37)	3.37 (2.33, 4.42)	2.51 (1.58, 3.45)
Quartile 3	−1.84 (−3.21, −0.46)	3.37 (2.33, 4.42)	2.71 (1.62, 3.80)
Quartile 4	−5.57 (−6.78, −4.36)	3.34 (2.24, 4.44)	3.27 (2.23, 4.30)
P for trend	<0.01	<0.01	0.01

Model 1: unadjusted. Model 2: adjusted for age, sex, and ethnicity. Model 3: adjusted for age, sex, ethnicity, marital status, alcohol consumption, hypertension, diabetes, special diet, total energy intake, coronary heart disease, and total cholesterol. *p < 0.05. BRI, Body Roundness Index; CI, Conicity Index; WHtR, waist-to-height ratio.

### 4.3 Smooth curve fitting and threshold effect analysis

**Figure 2:**
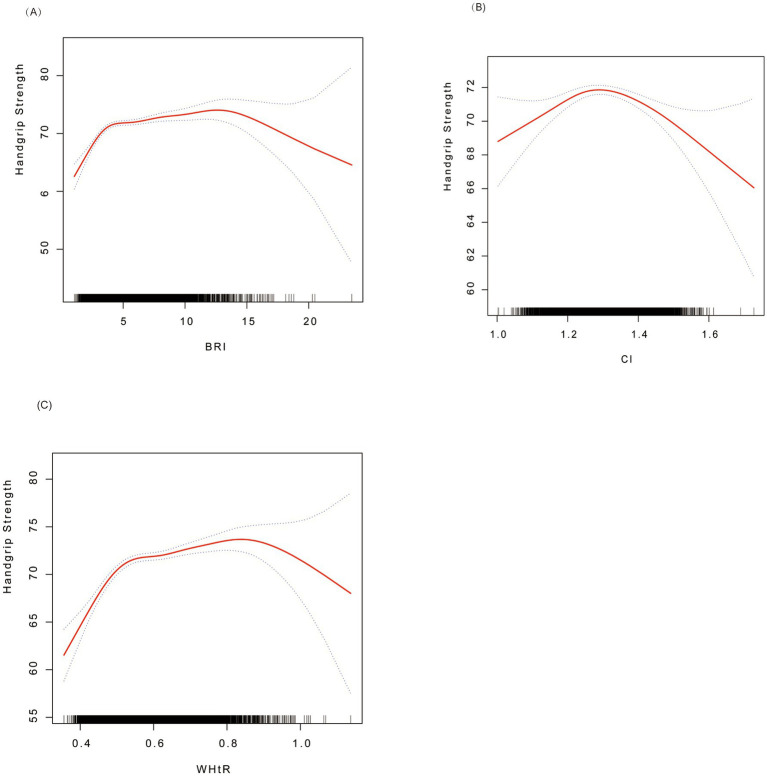
Smoothed curve fitting for associations of BRI, CI, and WHtR with handgrip strength. **(A)** BRI vs. grip strength; **(B)** CI vs. grip strength; **(C)** WHtR vs. grip strength. Red solid lines indicate smoothed curves, and the shaded areas represent 95% confidence intervals. Analyses were adjusted for age, sex, ethnicity, marital status, alcohol consumption, hypertension, diabetes, special diet, total energy intake, coronary heart disease, and total cholesterol. BRI, Body Roundness Index; CI, Conicity Index; WHtR, waist-to-height ratio.

Figure 2 shows the non-linear associations between BRI, CI, and WHtR and grip strength through smooth curve fitting. For BRI (Figure 2A), the grip strength increased rapidly at lower values (BRI < 3.55; *β* = 3.60, 95% CI: 2.81–4.39) and tended to level off at higher values (BRI ≥ 3.55; *β* = 0.24, 95% CI: 0.10–0.39) CI (Figure 2B) presented an inverted U-shaped relationship, and the grip strength reached its peak at the threshold of 1.27 (below the threshold: *β* = 15.87, 95% CI: 7.85–23.90; above the threshold: *β* = −9.98, 95% CI: −14.98−−4.98). WHtR (Figure 2C) showed an approximately linear positive correlation, with a stronger effect observed when it was below 0.51 (*β* = 62.46, 95% CI: 48.36–76.55), and the effect weakened but remained significant when it was above this threshold (*β* = 6.47, 95% CI: 2.85–10.08). All the inflection points were statistically significant (*p* < 0.01).

The threshold effect analysis (Table 3) demonstrated that BRI, CI, and WHtR had non-linear associations with grip strength, with the inflection points (K) identified at 3.55, 1.27, and 0.51, respectively. When the indicators were below the thresholds, each one-unit increase in BRI was associated with a significant increase in grip strength (*β* = 3.60, 95% CI: 2.81–4.39). CI (*β* = 15.87, 95% CI: 7.85–23.90) and WHtR (*β* = 62.46, 95% CI: 48.36–76.55) also showed significant positive effects (all *p* < 0.01). When the indicators were above the thresholds, the effects of BRI (*β* = 0.24, 95% CI: 0.10–0.39) and WHtR (*β* = 6.47, 95% CI: 2.85–10.08) weakened but still remained positively correlated, while CI turned into a negative correlation (*β* = −9.98, 95% CI: −14.98−−4.98). The above results indicate that BRI and WHtR continued to have a continuous positive effect on grip strength at high levels, although the effect was weakened. In contrast, CI showed a negative relationship with grip strength beyond the inflection point.

**Table 3:** Analysis of threshold effects between the three indicators and muscle strength.

Characteristic	BRI	CI	WHtR
Break point (K)	3.55	1.27	0.51
< K-segment effect	3.60 (2.81, 4.39)	15.87 (7.85, 23.90)	62.46 (48.36, 76.55)
> K-segment effect	0.24 (0.10, 0.39)	−9.98 (−14.98, −4.98)	6.47 (2.85, 10.08)
Log-likelihood ratio test	<0.01	<0.01	<0.01

Analyses were adjusted for age, sex, ethnicity, marital status, alcohol consumption, hypertension, diabetes, special diet, total energy intake, coronary heart disease, and total cholesterol. *P < 0.05. BRI, Body Roundness Index; CI, Conicity Index; WHtR, waist-to-height ratio.

### 4.4 Subgroup analysis

A subgroup analysis (Table 4) revealed that the associations between BRI, CI, and WHtR and grip strength were heterogeneous among different subgroups. BRI showed a consistent positive correlation in most subgroups (for example, among female individuals: *β* = 0.51, 95% CI: 0.36–0.65; among non-Hispanic Black individuals: *β* = 0.74, 95% CI: 0.50–0.99), and there were significant interactions with age (*p* = 0.01) and ethnicity (*p* = 0.01). CI exhibited differences in direction: it was negatively correlated among male individuals (*β* = −17.70, 95% CI: −24.59−−10.80) and the elderly population (*β* = −14.65, 95% CI: −20.80−−8.49) but positively correlated among female individuals (*β* = 6.29, 95% CI: 2.01–10.57) and the young population (*β* = 2.58, 95% CI: −3.38–8.54) (the interaction *p* < 0.01). WHtR maintained a positive association in most subgroups (for example, among non-drinkers: *β* = 15.72, 95% CI: 11.27–20.17; among the high-energy intake group: *β* = 8.57, 95% CI: 1.87–15.27) and showed significant associations with age, ethnicity, and drinking status (all *p* = 0.01).

**Table 4:** Subgroup analysis of associations between BRI, CI, and WHtR and grip strength.

Subgroup	BRI [*β* (95% CI)]	CI [*β* (95% CI)]	WHtR [*β* (95% CI)]
Sex: male	0.17 (−0.17, 0.52)	−17.70 (−24.59, −10.80)	4.93 (−3.07, 12.93)
Sex: female	0.51 (0.36, 0.65)	6.29 (2.01, 10.57)	13.30 (10.06, 16.55)
P for interaction	0.11	<0.01	0.08
Age: 20–39 years	0.56 (0.32, 0.80)	2.58 (−3.38, 8.54)	14.32 (8.78, 19.87)
Age: 40–60 years	0.12 (−0.07, 0.31)	−8.61 (−13.62, −3.59)	3.53 (−1.03, 8.09)
Age: >60 years	0.48 (0.26, 0.70)	−14.65 (−20.80, −8.49)	11.89 (6.80, 16.97)
P for interaction	0.01	<0.01	0.01
Ethnicity: Mexican American people	0.43 (0.05, 0.82)	−0.01 (−11.10, 11.08)	10.25 (0.60, 19.91)
Ethnicity: other Hispanic people	0.56 (−0.02, 1.14)	−5.60 (−20.64, 9.45)	13.63 (−0.35, 27.62)
Ethnicity: Non-Hispanic White people	0.23 (0.06, 0.39)	−7.53 (−12.14, −2.91)	6.20 (2.17, 10.22)
Ethnicity: Non-Hispanic Black people	0.74 (0.50, 0.99)	1.21 (−6.30, 8.71)	19.35 (13.31, 25.38)
Ethnicity: other ethnicities- including multiple ethnicities	0.90 (0.36, 1.45)	−0.91 (−16.48, 14.65)	22.28 (9.80, 34.76)
P for interaction	0.01	0.18	0.01
Education: less than 9th grade	0.08 (−0.34, 0.50)	−15.08 (−31.43, 1.27)	2.31 (−7.74, 12.35)
Education: 9–11th grade (includes 12th grade with no diploma)	0.39 (−0.07, 0.85)	−9.51 (−23.04, 4.03)	9.74 (−1.12, 20.60)
Education: high school graduate/GED or equivalent	0.46 (0.26, 0.66)	1.42 (−4.98, 7.83)	12.17 (7.08, 17.26)
Education: some college or AA degree	0.27 (0.03, 0.52)	−7.03 (−13.65, −0.40)	7.69 (1.70, 13.67)
Education: college graduate or above	0.52 (0.24, 0.80)	−5.05 (−11.70, 1.60)	12.32 (5.86, 18.78)
P for interaction	0.22	0.09	0.22
Marital status: married	0.30 (0.09, 0.52)	−5.74 (−12.29, 0.81)	8.01 (2.96, 13.06)
Marital status: widowed	1.16 (0.75, 1.57)	4.51 (−10.60, 19.62)	28.12 (17.72, 38.52)
Marital status: divorced	0.14 (−0.38, 0.66)	−9.43 (−22.28, 3.43)	4.24 (−8.18, 16.67)
Marital status: separated	0.46 (−0.14, 1.06)	−1.91 (−20.76, 16.95)	12.86 (−2.31, 28.04)
Marital status: never married	0.48 (0.20, 0.76)	−4.35 (−12.43, 3.73)	12.63 (5.77, 19.50)
P for interaction	0.01	0.65	0.01
drinking	0.32 (0.16, 0.47)	−6.73 (−10.91, −2.55)	8.32 (4.66, 11.98)
Non-drinking	0.60 (0.41, 0.78)	0.80 (−4.58, 6.17)	15.72 (11.27, 20.17)
P for interaction	0.01	0.02	0.01
Hypertension	0.27 (0.02, 0.51)	−8.55 (−17.12, 0.02)	7.38 (0.91, 13.85)
Non-hypertension	0.45 (0.27, 0.62)	−3.78 (−8.41, 0.86)	11.17 (7.11, 15.24)
P for interaction	0.27	0.37	0.36
Diabetes	0.39 (−0.00, 0.79)	−6.62 (−17.00, 3.75)	11.59 (2.26, 20.92)
Non-diabetes	0.38 (0.22, 0.53)	−5.04 (−9.62, −0.46)	9.63 (5.98, 13.28)
P for interaction	0.94	0.81	0.71
Coronary heart disease	−0.04 (−0.89, 0.82)	−22.51 (−46.70, 1.68)	−2.02 (−23.43, 19.40)
Non-coronary heart disease	0.39 (0.26, 0.53)	−4.66 (−8.69, −0.62)	10.27 (7.00, 13.55)
P for interaction	0.33	0.18	0.27
Other special diets	0.36 (0.03, 0.69)	−5.35 (−14.80, 4.10)	8.56 (0.54, 16.58)
Non-other special diets	0.38 (0.23, 0.54)	−5.20 (−9.21, −1.18)	10.14 (6.43, 13.84)
P for interaction	0.90	0.98	0.74
Low energy (kcal)	0.50 (0.25, 0.75)	−0.39 (−6.93, 6.15)	12.92 (7.10, 18.74)
Middle energy (kcal)	0.32 (0.08, 0.55)	−7.98 (−14.02, −1.93)	8.58 (2.83, 14.33)
High energy (kcal)	0.33 (0.05, 0.61)	−6.88 (−14.26, 0.50)	8.57 (1.87, 15.27)
P for interaction	0.56	0.27	0.56
Low total cholesterol (mmol/L)	0.43 (0.16, 0.71)	−8.00 (−15.03, −0.97)	10.87 (4.24, 17.50)
Middle total cholesterol (mmol/L)	0.37 (0.10, 0.64)	−2.63 (−9.92, 4.65)	9.90 (3.36, 16.44)
High total cholesterol (mmol/L)	0.32 (0.08, 0.57)	−4.93 (−11.83, 1.98)	8.66 (2.89, 14.42)
P for interaction	0.88	0.60	0.91

Analyses were adjusted for age, sex, ethnicity, marital status, alcohol consumption, hypertension, diabetes, special diet, total energy intake, coronary heart disease, and total cholesterol. *P*-values for interaction are shown below each subgroup. *P < 0.05. BRI, Body Roundness Index; CI, Conicity Index; WHtR, waist-to-height ratio.

## 5 Discussion

This study is the first to systematically reveal non-linear associations and threshold effects between BRI, CI, and WHtR and grip strength using a nationally representative U. S. cohort (NHANES 2011–2014). The results demonstrated that BRI and WHtR exhibited strong positive correlations with grip strength below specific thresholds (3.55 and 0.51, respectively), while CI displayed an inverted U-shaped relationship, shifting to a negative association beyond the threshold (1.27). These findings provide a novel perspective for sarcopenia screening, surpassing the limitations of traditional BMI in assessing muscle function.

Obesity and sarcopenia are two seemingly paradoxical, yet often coexisting metabolic disorders. With the intensification of global aging and shifts in lifestyle patterns, the prevalence of both conditions has increased markedly among older adults and is increasingly observed in younger populations (25). Studies suggest that obesity may directly or indirectly accelerate muscle loss through mechanisms such as chronic inflammation, insulin resistance, and aberrant adipose tissue deposition, culminating in the development of sarcopenic obesity (26). This complex condition not only exacerbates metabolic dysregulation but is also associated with elevated risks of disability and all-cause mortality (27).

From a pathophysiological perspective, the interplay between obesity and sarcopenia exhibits multidimensional characteristics. Adipose tissue is not merely an inert energy reservoir; its excessive expansion triggers the release of pro-inflammatory cytokines (e.g., TNF-*α* and IL-6). IL-6 suppresses mTOR via the JAK-STAT3 signaling pathway, thereby reducing protein synthesis, while concurrently activating NF-κB and FoxO3a. These factors promote the ubiquitin–proteasome system (UPS) and autophagy-lysosome pathway (ALP), accelerating muscle protein degradation. NF-κB further mediates TNF-α-induced protein loss in the skeletal muscle (28–30). Concurrently, obesity-associated insulin resistance inhibits the mTOR signaling pathway, diminishes muscle anabolic capacity, and drives ectopic lipid deposition within muscle tissue, forming intramuscular lipids (IMCLs) (31, 32). Adipose-derived dysregulation not only disrupts mitochondrial oxidative metabolism but also directly impairs muscle contraction efficiency (33). Abnormal glucose metabolism limits energy supply to muscles, ultimately leading to muscle degeneration and accelerated atrophy (34, 35). Additionally, reduced physical activity, leptin resistance, and diminished growth hormone secretion in individuals with obesity further destabilize the equilibrium between muscle synthesis and catabolism (36–38).

BMI fails to differentiate between fat and muscle mass (e.g., high BMI may reflect muscularity rather than obesity), leading to its paradoxical positive correlation with grip strength. In contrast, waist circumference, while partially reflecting abdominal adiposity, neglects height proportionality and geometric fat distribution patterns, resulting in its inverse association with muscle strength (12). By introducing BRI and WHtR, this study validated their ability to integrate waist circumference and height parameters, thereby more precisely quantifying visceral adipose tissue volume and circumventing the physiological limitations of conventional indices (14, 17). Within threshold ranges (BRI < 3.55; WHtR <0.51), the strong positive associations between BRI and WHtR and grip strength (*β* = 3.60 and 62.46, respectively) suggest that moderate visceral fat may support muscle function via energy reserves or adiponectin secretion (39, 40). Beyond these thresholds, the attenuated effects align closely with lipotoxicity, such as IL-6-mediated inflammatory responses (41).

Second, in validating non-linear associations, while Zhang et al. (13) reported a linear relationship between WHtR and metabolic abnormalities, our threshold model revealed a “piecewise linear” association for WHtR with grip strength (*β* = 62.46 below the threshold of 0.51), suggesting a saturation effect in the promotion of muscle function by fat distribution. Similarly, Lyu et al. (42) identified a positive correlation between BRI and grip strength in older adults, consistent with our findings. Our study further demonstrated a saturation effect between these variables. Regarding threshold generalizability, Ashwell et al. (17) proposed WHtR ≥0.5 as a cardiovascular risk threshold, whereas our study pinpointed WHtR = 0.51 as the inflection point for muscle function, indicating potential divergence in threshold values across distinct health outcomes. Notably, Baveicy et al. (15) reported a higher BRI threshold (≥4.2 in males) in Middle Eastern populations compared to our threshold of 3.55, highlighting the influence of ethnic morphological differences. Future studies are warranted to validate these thresholds in Asian and other populations.

This study identified significant age-, sex-, and ethnicity-dependent heterogeneity in the associations between BRI, CI, and WHtR and grip strength. For instance, CI exhibited a negative correlation in male individuals (*β* = −17.70, 95% CI: −24.59 to −10.80) but a positive correlation in female individuals (*β* = 6.29, 95% CI: 2.01–10.57), which may be attributed to estrogen-mediated regulation of fat distribution (43). Additionally, the strongest BRI effect was observed in non-Hispanic Black individuals (*β* = 0.74, 95% CI: 0.50–0.99), suggesting that genetic or lifestyle factors may modulate adipose-muscle crosstalk (44, 45). These findings underscore the necessity of incorporating individual characteristics (e.g., sex and ethnicity) into clinical evaluations to optimize the selection of obesity indices for muscle health assessment.

From a clinical perspective, the thresholds of BRI (3.55) and WHtR (0.51) may serve as screening references for muscle strength abnormalities, particularly in individuals with central obesity. For example, even if grip strength falls within the normal range, those with BRI > 3.55 should be monitored for risks of compensatory decline in muscle function. Subgroup-specific strategies are warranted: older male individuals require focused surveillance for muscle loss when CI exceeds 1.27, while non-Hispanic Black populations may prioritize BRI trajectories to evaluate intervention efficacy. At the public health level, it is recommended to integrate these indices into the community health assessment systems, coupled with grip strength measurements, to optimize early identification protocols for sarcopenia.

Future research should adopt multidisciplinary approaches to deepen exploration in the following directions. Longitudinal study designs could track temporal changes in fat distribution and muscle strength, thereby clarifying causal relationships. The integration of imaging techniques, such as MRI and DXA, would enable the direct quantification of visceral adipose tissue and muscle mass, reducing indirect measurement biases associated with anthropometric proxies. Expanding sample diversity—particularly incorporating Asian and African populations—is essential to validate the generalizability of thresholds across ethnic groups. Additionally, incorporating data from exercise or nutritional interventions will help unravel dynamic regulatory mechanisms between adiposity indices and muscle strength. Collectively, these advancements will advance precision screening for sarcopenia and support the development of personalized health management strategies tailored to individual metabolic and morphological profiles.

## 6 Conclusion

In U. S. adults, BRI, CI, and WHtR exhibit non-linear, threshold-dependent associations with grip strength, demonstrating significant evaluative utility, particularly among populations with central obesity. Future longitudinal studies are warranted to validate causal relationships and establish these indices as novel biomarkers for the precision screening of sarcopenia.

## Data Availability

The original contributions presented in the study are included in the article/supplementary material, further inquiries can be directed to the corresponding author.

## Footnotes

^1^ https://www.cdc.gov/nchs/nhanes/index.html

^2^ www.empowerstats.com
